# Nitrification Progress of Nitrogen-Rich Heterocyclic Energetic Compounds: A Review

**DOI:** 10.3390/molecules27051465

**Published:** 2022-02-22

**Authors:** Yiming Luo, Wanwan Zheng, Xuanjun Wang, Fei Shen

**Affiliations:** 1 High-Tech Institute of Xi’an, Xi’an 710025, China; iamrlym@126.com (Y.L.); shenf02@163.com (F.S.); 2 Xi’an Modern Chemistry Research Institute, Xi’an 710065, China; 3 School of Chemical Engineering, Northwest University, Xi’an 710069, China; zhengwan2020@163.com

**Keywords:** nitro group, nitration, nitrogen rich heterocycle, energetic materials

## Abstract

As a momentous energetic group, a nitro group widely exists in high-energy-density materials (HEDMs), such as trinitrotoluene (TNT), 1,3,5-triamino-2,4,6-trinitrobenzene (TATB), cyclo-1,3,5-trimethylene-2,4,6-trinitramine (RDX), etc. The nitro group has a significant effect on improving the oxygen balance and detonation performances of energetic materials (EMs). Moreover, the nitro group is a strong electron-withdrawing group, and it can increase the acidity of the acidic hydrogen-containing nitrogen-rich energetic compounds to facilitate the construction of energetic ionic salts. Thus, it is possible to design nitro-nitrogen-rich energetic compounds with adjustable properties. In this paper, the nitration methods of azoles, including imidazole, pyrazole, triazole, tetrazole, and oxadiazole, as well as azines, including pyrazine, pyridazine, triazine, and tetrazine, have been concluded. Furthermore, the prospect of the future development of nitrogen-rich heterocyclic energetic compounds has been stated, so as to provide references for researchers who are engaged in the synthesis of EMs.

## 1. Introduction

Nitrogen-rich compounds have attracted widespread attention in energetic materials (EMs) because of their advantages of outstanding density, excellent positive enthalpy of formation, remarkable detonation performance, and high thermal stability [1,2,3]. They can be used in explosives, propellants, gas generators, and smokeless pyrotechnic fuels [4,5]. In order to meet the increasing performance requirements, new nitrogen-rich EMs are being developed with upgradable density, better detonation performance, lower impact and friction sensitivity, and higher thermal stability.

One of the most popular strategies for the design of promising new EMs is the incorporation of both fuel and oxidizer moieties into one molecule. [6,7] The nitro group is a pivotal explosive group, which exists in trinitrotoluene (TNT), 1,3,5-triamino-2,4,6-trinitrobenzene (TATB), cyclo-1,3,5-trimethylene-2,4,6-trinitramine (RDX), cyclo-1,3,5-7-tetramethylene-2,4,6,8-tetranitr-amine (HMX), and hexanitrohexaazaisowurtzitane (CL-20), etc. Nitro groups in the molecular structure can heighten the oxygen balance, increase the density, and significantly enhance the detonation performance of EMs [8,9]. Moreover, the nitro group is a strong electron-withdrawing group, and it can increase the acidity of hydrogen-containing nitrogen-rich energetic compounds and is conducive to the construction of energetic ionic salts [10]. Therefore, this paper reviews the nitrification methods for nitrogen-rich heterocyclic energetic compounds so as to give some suggestions for this special reaction.

The main skeletons of nitrogen-rich heterocyclic energetic compounds are azole rings (imidazole, pyrazole, triazole, tetrazole, oxadiazole) and azine rings (pyrazine, pyridazine, triazine, tetrazine) [11,12]. Different nitration systems can be used to nitrate specific compounds on the basis of the structural characteristics of compounds [13]. According to the diversity of the nitration positions on these frameworks, this paper classifies the nitration of H on heterocyclic C, the nitration of -NH_2_ on heterocyclic C, the nitration of H on heterocyclic N, and the nitration of -NH_2_ on heterocyclic N. 

## 2. Nitrification of Azoles Nitrogen-Rich Heterocyclic Energetic Compounds

### 2.1. Imidazoles

#### 2.1.1. Nitrification of H on Imidazole Ring C

4,4′,5,5′-tetranitro-2,2′-benzimidazole (TNBI) is an important precursor for the synthesis of EMs. It has been synthesized by two nitrification systems.

(1) NaNO_3_/H_2_SO_4_ system

**Scheme 1:**
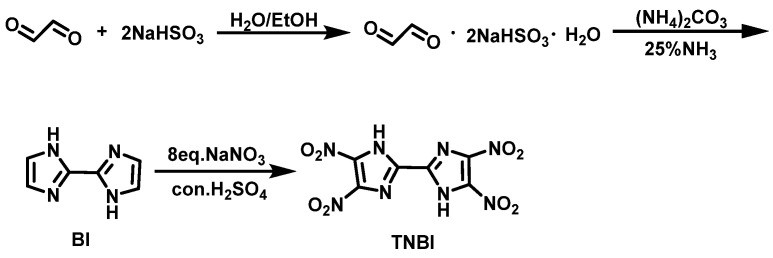
Synthesis of 4,4′,5,5′-tetranitro-2,2′-biimidazole [14].

Klapötke et al. [14] synthesized 2,2′-biimidazole (BI) from glyoxal and sodium bisulfite by cyclization, suspended NaNO_3_ and urea in concentrated H_2_SO_4_ (96–98%) at 0 °C, and added BI in small batches. The suspension was stirred for 1 h at ambient temperature, and then heated to 85–90 °C for 16 h. Thereafter, the suspension was poured onto crushed ice, filtered, and washed with ice water to obtain TNBI·2H_2_O, with a yield of 51%. The synthesis route is shown in Scheme 1.

(2) 80% HNO_3_/H_2_SO_4_ system

**Scheme 2:**
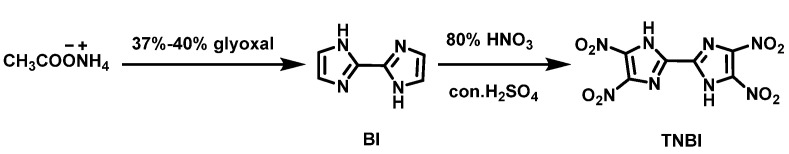
Synthesis of 4,4′,5,5′-tetranitro-2,2′-biimidazole [15].

Li et al. [15] synthesized BI using ammonium acetate (CH_3_COONH_4_) and glyoxal as raw materials. Then, BI was added to 95–98% concentrated H_2_SO_4_ at 20–25 °C. After that, the mixed solution of 80% HNO_3_ and 95–98% H_2_SO_4_ was added dropwise as the mixture was heated to 45 °C. Four hours later, the reaction solution was poured onto crushed ice, filtered, washed with cold water, and dried to obtain TNBI. The synthesis route is shown in Scheme 2. The yield was 51.7%. Compared with the traditional operation process [14], this strategy saves the reaction time and lowers the reaction temperature via a gentle and stable reaction process, thus, the danger of the reaction is reduced.

#### 2.1.2. Nitration of -NH_2_ on Imidazole Ring C/N

The H atom of NH_2_ can be nitrified to obtain different nitration products using the HNO_3_/H_2_SO_4_ system.

**Scheme 3:**
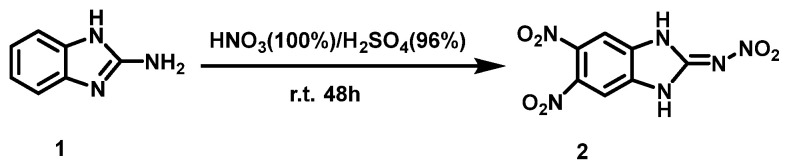
Synthesis of 2-nitrimino-5,6-dinitrobenzimidazole [16].

Thomas et al. [16] added 2-aminobenzimidazole (1) to the solution of 100% HNO_3_ and concentrated H_2_SO_4_ (96–98%) while stirring at 0 °C. After stirring for 48 h at 25 °C, the mixture was poured onto crushed ice, then filtered and washed with 20% HNO_3_ and a small amount of water. The light yellow solid, 2-nitroammonium-5,6-dinitrobenz imidazole (2), was obtained with a yield of 41%. The synthesis route is shown in Scheme 3.

**Scheme 4:**
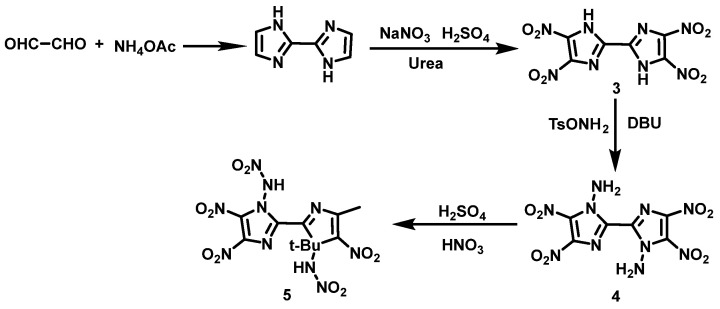
Synthesis of *N*,*N*′-dinitramino-4,4′,5,5′-tetranitro-bisimidazole [17].

Another example is nitration of -NH_2_ on imidazole ring N. Yin et al. [17] synthesized 4,4′,5,5′-tetranitro-1*H*,1′*H*-(2,2′-benzimidazole)-1,1′-diamine (4) from glyoxal and ammonium acetate by using condensation, nitration, and N-amination reactions. Compound **4** was then slowly added to the HNO_3_/H_2_SO_4_ solution at −10 °C and stirred for 90 min. The solution was subsequently poured onto crushed ice, stirred for about 10–15 min, filtered, and washed with ice-cooled water, ethanol, and ether to obtain *N*,*N*′-dinitroamino-4,4′,5,5′-tetranitro-bisimidazole (5). The synthesis route is shown in Scheme 4. In order to prevent the N-N bond from being broken, the nitration reaction should be performed in a mixed acid at a temperature of −15 to −10 °C because the N-amino group is highly reactive. It has been found that the nitrification ability of HNO_3_/H_2_SO_4_ is stronger than that of HNO_3_, so HNO_3_/H_2_SO_4_ with an appropriate ratio is always selected for nitrating NH_2_ to NHNO_2_. The reaction condition is mostly mild and the operation is simple.

#### 2.1.3. Nitrification of H on Imidazole Ring N

(1) HNO_3_/Ac_2_O system

**Scheme 5:**
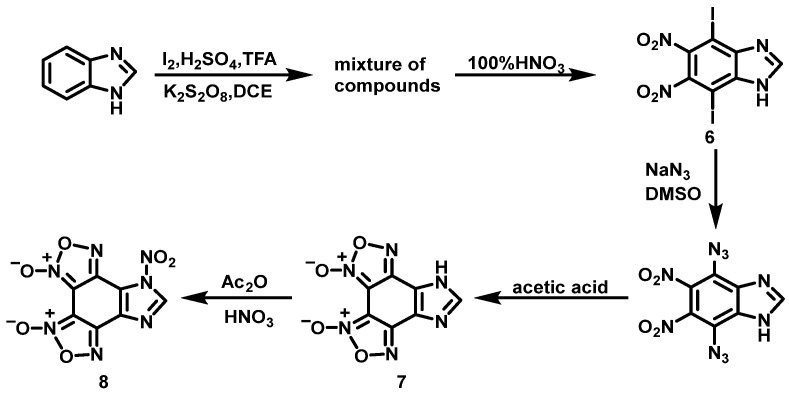
Synthesis of 7-nitro-7*H*-imidazo[4′,5,5,6]benzo[1,2,3,4]bis([1,2,5]oxadiazole)-3,4-dioxide [18].

Chand et al. [18] synthesized compound **7** using benzimidazole as a raw material through iodination, nitration, substitution, and ring formation reactions (Scheme 5). Then, 100% HNO_3_ was added dropwise to acetic anhydride (Ac_2_O) at −5 °C, and the resulting mixture was stirred for 0.5 h. Compound **7** was then added to the reaction solution in portions and stirred for 2 h. Afterwards, the reaction mixture was poured onto crushed ice. Compound **8** was obtained by filtration with a yield of 67%. Compared with the nitrifying agent HNO_3_/H_2_SO_4_, the nitrifying ability of HNO_3_/Ac_2_O mixed solution is weaker, but Ac_2_O can effectively decrease the oxidizing property of HNO_3_ and avoid the formation of by-products of compound **8**.

### 2.2. Nitrification of Pyrazoles

#### 2.2.1. Nitrification of H on Pyrazole Ring C

(1) Bismuth nitrate/montmorillonite

**Scheme 6:**
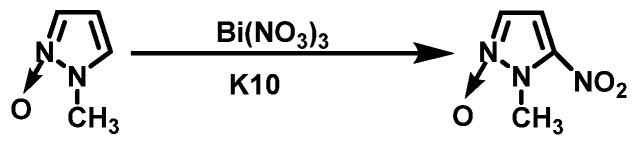
Synthesis of 1-methyl-5-nitropyrazole- 2-oxide (K-10) [19].

P. Ravi et al. [19] added 1-methylpyrazole-2-oxide and montmorillonite (K-10) to a suspension of bismuth nitrate (Bi(NO_3_)_3_) in tetrahydrofuran (THF) and stirred for 2.5 h. The solvent was then evaporated under reduced pressure using a vacuum pump for 5 min. The mixture was repeatedly washed with dichloromethane (CH_2_Cl_2_) and concentrated to give the crude K-10. The pure product was isolated by column chromatography with a yield of 98%. The synthesis route is shown in Scheme 6. The ranking of nitration capacities of some nitrates is as follows: Bi(NO_3_)_3_ > AgNO_3_ > KNO_3_ > NaNO_3_ > NH_4_NO_3_ > Pb(NO_3_)_2_ > Ba(NO_3_)_2_. In the reaction, Bi(NO_3_)_3_, which is impregnated on K-10, has a fast nitration rate and high yield and is easy to separate from the product by filtration. The methyl group in the pyrazole ring contributes to the nitration process, and the nitration rate will increase with the number of methyl groups.

(2) HNO_3_/H_2_SO_4_ system

**Scheme 7:**
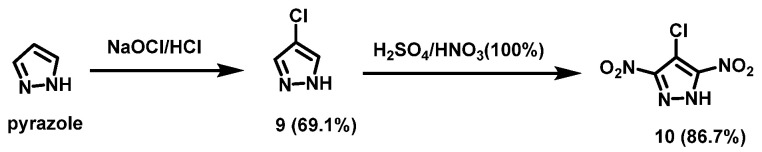
Synthesis of 4-chloro-3,5-dinitropyrazole [20].

The HNO_3_/H_2_SO_4_ system usually has a strong nitration effect. Fischer et al. [20] synthesized 4-chloropyrazole (9) using pyrazole as a raw material via chlorination reaction. Compound **9** was then dissolved in the concentrated H_2_SO_4_, and 100% HNO_3_ was slowly added below 40 °C. Then, the mixture was heated to 100 °C and stirred for 5 h under reflux. After cooling to room temperature, the final solution was poured onto crushed ice water and extracted with acetoacetic acid. The organic layers were washed with water, dried over magnesium sulfate, and dried under nitrogen to give 4-chloro-3,5-dinitro-1*H*-pyrazole (10) in a yield of 86.7%. The synthesis route is shown in Scheme 7. The reaction is simple, no further purification is required, and the reaction yield is high.

(3) HNO_3_/P_2_O_5_ system

**Scheme 8:**
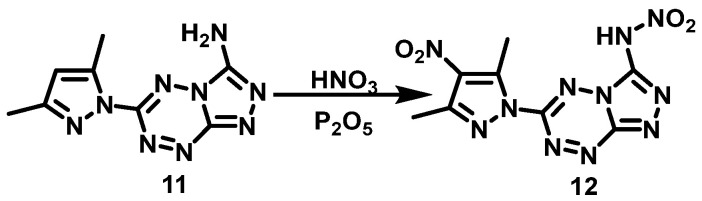
Synthesis of *N*-(6-(3,5-dimethyl-4-nitro-1*H*-pyrazol-1-yl) -1,2,4-triazolo[4,3-b]-1,2,4,5-tetrazine-3-yl)nitramide [21].

Wang et al. [21] dissolved P_2_O_5_ in fuming HNO_3_ and then added (6-(3,5-dimethyl-1*H*- pyrazole-1-yl)-1,2,4-triazole[4,3-b]-1,2,4,5-tetrazine-3-amino (11) at 0 °C. The reaction mixture was stirred for 10 h at room temperature. Then, the mixture was subsequently poured onto crushed ice, extracted with ethyl acetate, and purified by column chromatography to obtain *N*-(6-(3,5-dimethyl- 4-nitro-1*H*-pyrazole-1-yl)-1,2,4-triazolo[4,3-b]-1,2,4,5-tetrazin-3-yl)nitramide (12) with a yield of 58%. In this reaction, the NH_2_ in 1,2,4-triazol ring is also nitrated to NHNO_2_. The synthesis route is shown in Scheme 8. The nitrification system used in this reaction is HNO_3_/P_2_O_5_. P_2_O_5_ is not only a dehydrating agent, but also a nitrification promoter. This nitration system is not only suitable for aromatics but also for amines. Even amines that are difficult to nitrate can get satisfactory results sometimes.

#### 2.2.2. Nitrification of -NH on Pyrazole Ring C

(1) 100% HNO_3_ system

**Scheme 9:**
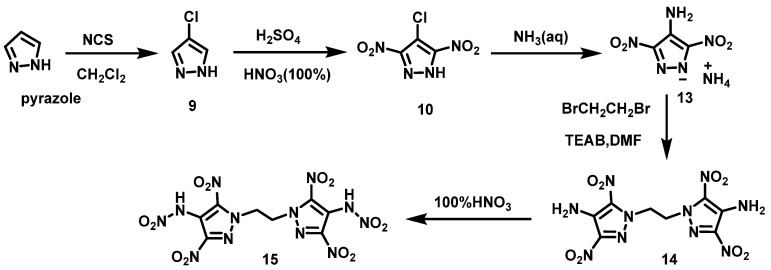
Synthesis of *N*,*N*′-[1,1′-(ethane-1,2-diyl)bis(3,5-dinitro-1*H*-pyrazole-4,1-diyl)]dinitramide [22].

HNO_3_ is a strong nitration agent, it can be used to nitrate NH_2_ to NHNO_2_. Yin et al. [22] synthesized 1,1′-(ethane-1,2-diyl)bis(3,5-dinitro-)1*H*-pyrazole-4-amine) (14) using pyrazole as raw material through halogenation, nitration, neutralization, and alkylation reaction. Afterwards, compound **14** was added to 100% HNO_3_ in portions below 10 °C. The reaction was held for 10 min at 5 °C, and HNO_3_ was removed by blowing in air. The residue was dried under vacuum to give *N*,*N*′-[1,1′-(ethane-1,2-diyl)bis(3,5-dinitro-1*H*-pyrazole-4,1-diyl)]dinitramide (15). The synthesis route is shown in Scheme 9. This process uses 100% HNO_3_ as the nitration system. For the nitration of different azole rings, different concentrations of HNO_3_ are required.

(2) 70% HNO_3_/H_2_SO_4_ system

**Scheme 10:**
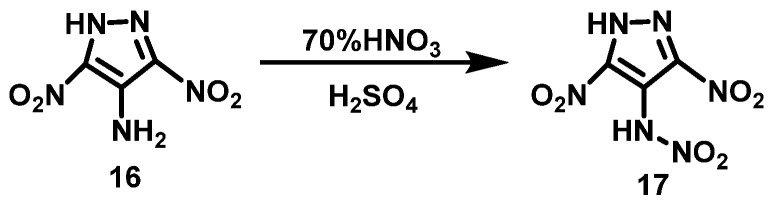
Synthesis of 4-nitramino-3,5-dinitropyrazole [23].

Zhang et al. [23] added 4-amino-3,5-dinitropyrazole (16) to a mixture of HNO_3_ (70%) and concentrated H_2_SO_4_ in a volume ratio of 1:1 at 0 °C. The mixture was stirred for 2 h and then slowly warmed to room temperature. After stirring for another 4 h, the reaction mixture was poured into ice water and extracted with ether to obtain 4-nitroamino-3,5-dinitropyrazole (17). The synthesis route is shown in Scheme 10.

(3) HNO_3_/Ac_2_O system

**Scheme 11:**
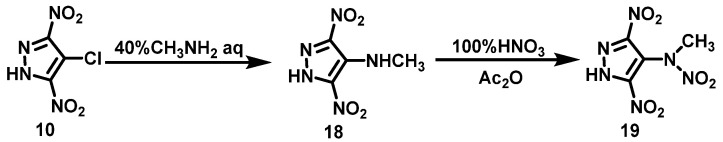
Synthesis of 4-(*N*-methylnitramino)-3,5-dinitropyrazole [24].

He et al. [23] synthesized 4-methylamino-3,5-dinitropyrazole (18) using 4-chloro-3,5-dinitro pyrazole (10) as a raw material through nucleophilic substitution reaction. Firstly, 100% HNO_3_ was slowly added to a cooled solution of compound **18** in acetic acid, after which Ac_2_O was added, and the mixture was stirred for 1.5 h at room temperature. 4-(*N*-methylnitramino)-3,5-dinitropyrazole (19) was finally obtained by removing excess acid under vacuum with a yield of 95%. The synthesis route is shown in Scheme 11. This reaction uses HNO_3_/Ac_2_O as a nitration system, which is simple to operate, has no by-products, and the yield is as high as 95%.

#### 2.2.3. Nitrification of H on Pyrazole Ring N

(1) HNO_3_/Ac_2_O system

**Scheme 12:**
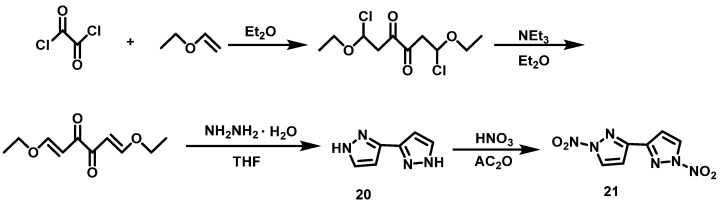
Synthesis of 1,1′-dinitro-1*H*,1*H*’-3,3′-bipyrazole [25].

Tang et al. [25] synthesized 1*H*,1′*H*-3,3′-bispyrazole(20) using oxalyl chloride and ethoxyethylene as raw materials through addition, elimination, and cyclization reactions. Firstly, 100% HNO_3_ was added dropwise to AC_2_O at 0 °C, then compound **20** was slowly added to the mixed acid. The reaction mixture was warmed to room temperature, and stirred for 6 h. The reaction mixture was subsequently poured into ice water, filtered, and washed with trifluoroacetic acid (TFAA) to obtain 1,1′-dinitro-1*H*, 1′*H*-3,3′-bispyrazole (21), with a yield of 71%. The synthesis route is shown in Scheme 12.

(2) NH_4_NO_3_/TFAA system

**Scheme 13:**
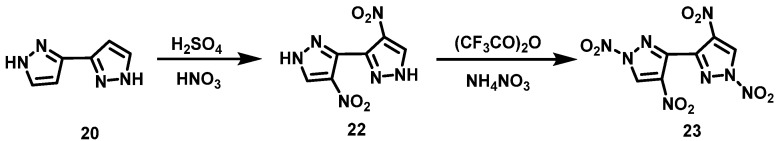
Synthesis of 4,4′,5,5′-tetranitro-2*H*,2′*H*-3,3′-bipyrazole [26].

Kumar et al. [26] prepared 4,4′-dinitro-1*H*,1′*H*-3,3′-bipyrazole (22) by nitrating bipyrazole (20) with nitric–sulfur mixed acid. Then, NH_4_NO_3_ was added to the suspension of compound **5** in TFAA ((CF_3_CO)_2_O) in batches at 0–5 °C. The reaction mixture was stirred for 5 h at room temperature, filtered, and washed with water to obtain 1,1′,4,4′-tetranitro-1*H*,1′*H*-3,3′-dipyrazole (23). The synthesis route is shown in Scheme 13. NH_4_NO_3_/TFAA are used as nitration reagents to make reaction conditions mild, and post-treatment of waste acid is not needed at the end of the reaction.

#### 2.2.4. Nitrification of -NH_2_ Connected with the Pyrazole Ring N

A fuming HNO_3_/con. H_2_SO_4_ system can be used to nitrate -NH_2_ to NHNO_2_, which is connected with N in a pyrazole ring.

**Scheme 14:**
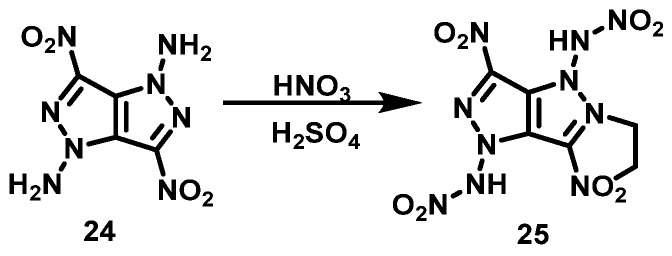
Synthesis of *N*,*N*′-(3,6-dinitropyrazole [4,3-c] pyrazole-1,4-diyl) dinitramine [27].

Yin et al. [27] cooled a concentrated H_2_SO_4_ suspension of 3,6-dinitropyrazole[4,3-c] pyrazole-1,4-diamine (24) to −15 °C in an ice-salt bath, then fuming HNO_3_ was added dropwise to the mixture. After the mixture was stirred for 2 h at −15 °C, *N*,*N*′-(3,6-dinitropyrazole[4,3-c] pyrazole-1,4-diyl)dinitramine (25) was obtained by filtering the reaction solution and washing with TFAA. The synthesis route is shown in Scheme 14.

### 2.3. Triazoles

#### 2.3.1. Nitrification of H on 1,2,4-Triazole Ring C

(1) 70% HNO_3_ system

**Scheme 15:**
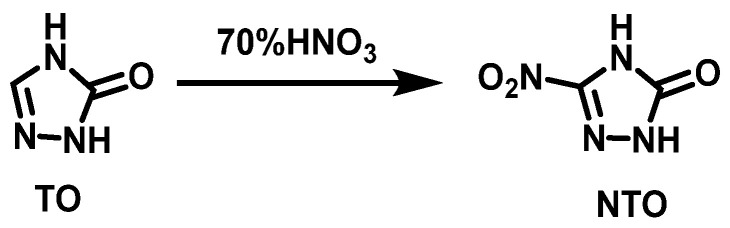
Synthesis of 3-nitro-1,2,4-triazol-5-one [30].

3-nitro-1,2,4-triazol-5-one (NTO) is a kind of insensitive high-energy explosive with excellent comprehensive performance [28,29]. The more mature synthesis method of NTO is by nitration of TO(1,2,4-triazol-5-one), which is synthesized from semicarbazide hydrochloride and formic acid using condensation and cyclization reaction. The synthesis route is shown in Scheme 15. The nitrating agent is 70% or 98% HNO_3_. Huang et al. [30] added TO to 70% HNO_3_ in batches at 60–65 °C, reacted for 1 h, then cooled to 3 °C in an ice-water bath, filtered, and collected the HNO_3_ filtrate, which was recycled in the next batch of reactions. The filter cake was rinsed with water and followed by vacuum filtration. Finally, NTO was obtained via recrystallization from water with a yield of 75.4% and a purity of 99.94%. Compared with the use of 98% HNO_3_ as the nitrating agent, this nitration method has a simpler process, safer operation, and HNO_3_ filtrate can be reused, so the cost of raw materials can be lowered.

(2) HNO_3_/Ac_2_O system

**Scheme 16:**
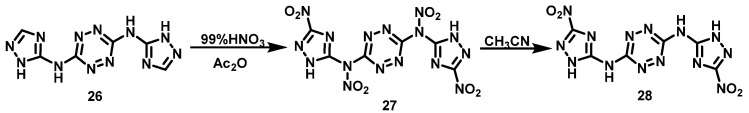
Synthesis of *N*^3^,*N*^6^-Bis(3-nitro-1H-1,2,4-triazol-5-yl)-1,2,4,5-tetrazine-3,6-diamine [31].

Aizikovich et al. [31] added 99% HNO_3_ to Ac_2_O at 0 °C while stirring for 30 min. The solid *N*^3^,*N*^6^-bis(1H-1,2,4-triazol-5-yl)-1,2,4,5-tetrazine-3,6-diamine (26) was slowly added to the mixture, after which, the solution was stirred for 2 h under anhydrous conditions. Then, the reaction solution was warmed to room temperature, filtered and washed quickly with TFAA, and immediately redissolved in hot CH_3_CN. The obtained CH_3_CN solution was heated for 30 min at 65 °C, then cooled to room temperature. Pure *N*^3^,*N*^6^-bis(3-nitro-1H-1,2,4-triazole-5-yl)-1,2,4,5-tetrazine-3,6-diamine (28) was filtrated and washed with CH_3_CN to give a yield of 31%. The synthesis route is shown in Scheme 16. Although the yield of the reaction is low, the operation is simple with short reaction time, and the product does not need further purification.

#### 2.3.2. Nitration of -NH_2_ on 1,2,4-Triazole Ring C

(1) HNO_3_/concentrated H_2_SO_4_ system

**Scheme 17:**
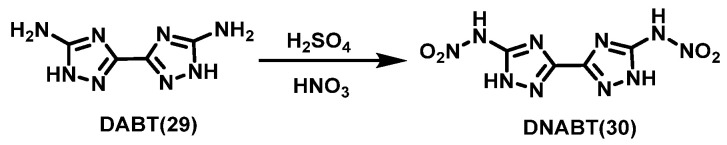
Synthesis of 3,3′-dinitrimino-5,5′-bis(1*H*-1,2,4-triazole) [33].

From the synthetic method by Astachov et al. [32], Dippold et al. [33] synthesized 3,3′-diamino-5,5′-bis (1*H*-1,2,4-triazole) (DABT,29) via condensation reaction using oxalic acid and aminoguanidine bicarbonate as raw materials. After that, HNO_3_ was slowly added to a concentrated H_2_SO_4_ solution of compound **29** at 0 °C. The mixture was warmed to room temperature and stirred for 1 h. The clear solution was then poured onto ice and the precipitate was collected by filtration. A yellow crystalline solid of 3,3′-dinitramine-5,5′-bis (1*H*-1,2,4-triazole) (DNABT, 30) was obtained by recrystallization from boiling water with a yield of 77%. The synthesis route is shown in Scheme 17.

(2) HNO_3_/P_2_O_5_ system

**Scheme 18:**
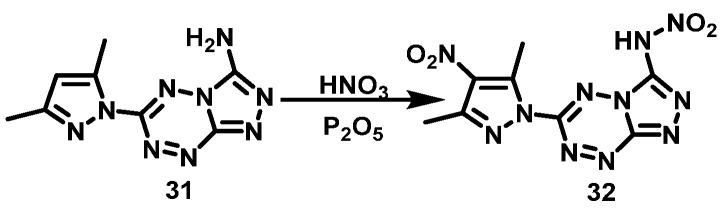
Synthesis of N-(6-(3,5-dimethyl-4-nitro-1H-pyrazol-1-yl) -1,2,4-triazolo[4,3-b]-1,2,4,5-tetrazin-3-yl)nitramide [21].

Wang et al. [21] dissolved P_2_O_5_ in fuming HNO_3_, then (6-(3,5-dimethyl-1*H*-pyrazol-1-yl)-1,2,4- triazole[4,3-b]-1,2,4,5-tetrazine-3-amino (31) was added to the mixture at 0 °C and stirred at room temperature for 10 h. Then, the mixture was poured onto crushed ice and extracted with ethyl acetate, after which, *N*-(6-(3,5-dimethyl-4-nitro-1*H*-pyrazol-1-yl)-1,2,4-triazole[4,3-b]-1,2,4,5-tetrazin-3-yl) nitramine (32) was purified by column chromatography with a yield of 58%. The synthesis route is shown in Scheme 18. The nitrating reagents used in this reaction are relatively mild, but the post-treatment is relatively complicated.

#### 2.3.3. Nitrification of H on 1,2,4-Triazole Ring N

(1) HNO_3_/Ac_2_O system

**Scheme 19:**
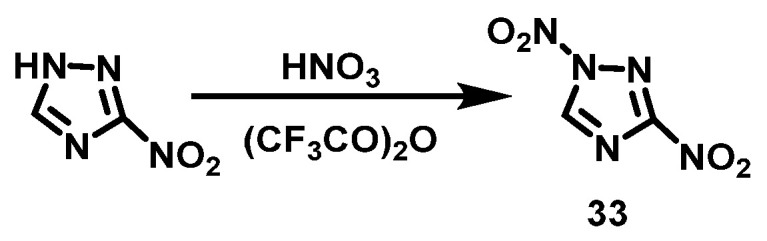
Synthesis of 1,3-dinitro-1*H*-1,2,4-triazole [34].

Yin et al. [34] dissolved TFAA and 3-nitro-1,2,4-triazole in chloroform at 0~5 °C, and fuming HNO_3_ was added dropwise while maintaining the temperature. After the dropwise addition was completed, the reaction was warmed and stirred for 1 h at room temperature. The reaction solution was then poured onto crushed ice, and 1,3-dinitro-1*H*-1,2,4-triazole (33) was obtained by extraction with chloroform with a yield of 53%. The synthesis route is shown in Scheme 19.

#### 2.3.4. Nitrification of -NH_2_ on 1,2,4-Triazole Ring N

(1) HNO_3_ system

**Scheme 20:**
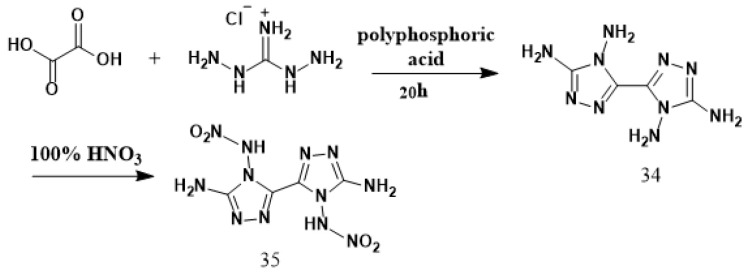
Synthesis of 5,5′-diamino-4,4′-dinitramino-3,3′-bi-1,2,4-triazole [35].

Klapçtke et al. [35] synthesized 4,4′,5,5′-tetraamino-3,3′-bi-1,2,4-triazole (34) using oxalic acid and diaminoguanidine hydrochloride as raw materials via a condensation reaction under the action of polyphosphoric acid. HNO_3_ was cooled to −10 °C and compound **1** was slowly added while the temperature was kept below 0 °C. After that, the mixture was stirred for 90 min at −5 °C. Then, the solution was poured onto crushed ice, stirred for 10–15 min, filtered, and 5,5′-diamino-4,4′-dinitroammonium-3,3′-di-(1,2,4-triazole) (35) was obtained by washing with cold water, ethanol, and ether, respectively, with a yield of 69%. The synthesis route is shown in Scheme 20. The synthetic method is simple in operation, and the obtained product does not require further purification.

#### 2.3.5. Nitration of -NH_2_ Connected with 1,2,3-Triazole Ring C

**Scheme 21:**
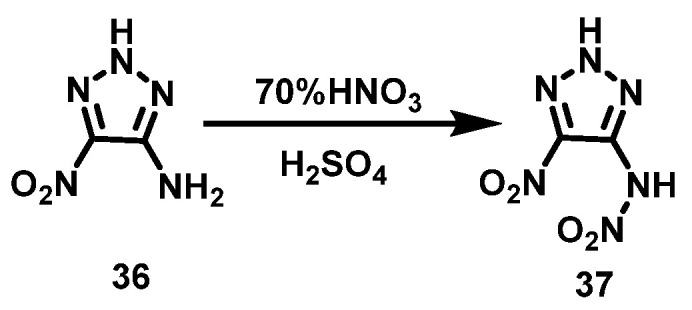
Synthesis of 4-nitramino-5-nitro-1,2,3-2*H*-triazole [36].

Zhang et al. [36] added 4-amino-5-nitro-1,2,3-2*H*-triazole (36) to a mixture of HNO_3_ (70%) and concentrated H_2_SO_4_ in batches and stirred for 2 h at 0 °C. After stirring for another 2 h at room temperature, the reaction mixture was poured into ice water. 4-nitramine-5-nitro-1,2,3-2*H*-triazole (37) was obtained by ether extraction, and the yield was 76%. The synthesis route is shown in Scheme 21.

#### 2.3.6. Nitrification of H on 1,2,3-Triazole Ring N

**Scheme 22:**
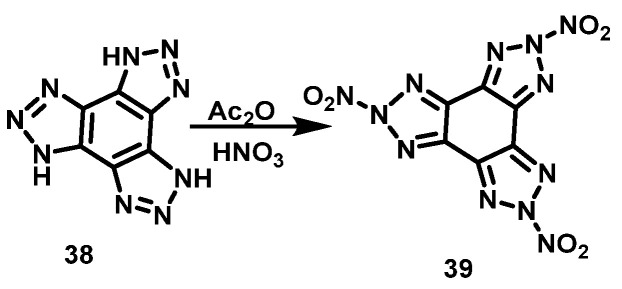
Synthesis of 2,5,8-trinitrotris(triazolo)benzene [37].

Thottempudi et al. [37] added concentrated HNO_3_ to Ac_2_O dropwise at −5 °C, the mixture was stirred for 30 min, and then stirred for another 45 min at room temperature. Subsequently, the mixture was cooled to −5 °C, and tris(triazolo) benzene (38) was added in portions, stirred for about 15 min, and stirred overnight at room temperature. The mixture was then poured onto ice, filtered, and washed with water to obtain the final product of trinitrotris(triazolo)benzene (39) with a yield of 53%. The synthesis route is shown in Scheme 22.

### 2.4. Tetrazoles

#### 2.4.1. Nitrification of -NH_2_ on Tetrazole C

(1) NO_2_BF_4_ system

**Scheme 23:**
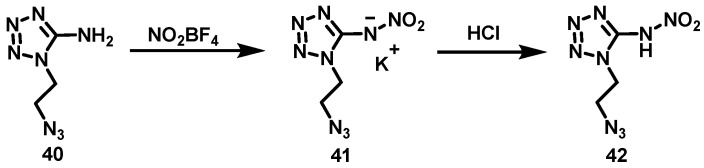
Synthesis of 1-(2-azidoethyl)-5-nitriminotetrazole [38].

Stierstorfer et al. [38] dissolved 1-(2-azidoethyl)-5-aminotetrazole (40) in MeCN in an ice-water bath, and added nitronium tetrafluoroborate (NO_2_BF_4_) to the solution. The solution was then stirred for 30 min at 0 °C and for another 1 h at 25 °C. The solvent was evaporated and KOH ethanol solution was added. The precipitated KBF_4_ was removed by filtration and another equivalent of KOH in ethanol was added. The solvent was evaporated and cold water was added. The insoluble material was removed by filtration and the water was evaporated. Potassium 1-(2-azidoethyl)-5-nitroaminotetrazole (41) was finally obtained by recrystallization from hot ethanol with a yield of 33%. After that, an equimolar amount of dilute (1N) HCl was added to compound **41**. Then, the solvent was evaporated and acetone was added to the residue. The solution was filtered to remove KCl. After acetone was evaporated, the crude product was recrystallized from a small amount of methanol to yield colorless 42 (90%). The synthesis route is shown in Scheme 23. The reaction conditions are mild and the temperature is easy to control. However, HBF_4_ must be removed after the reaction, the yield of compound **41** is low, and the purification is difficult.

(2) N_2_O_5_ system

**Scheme 24:**
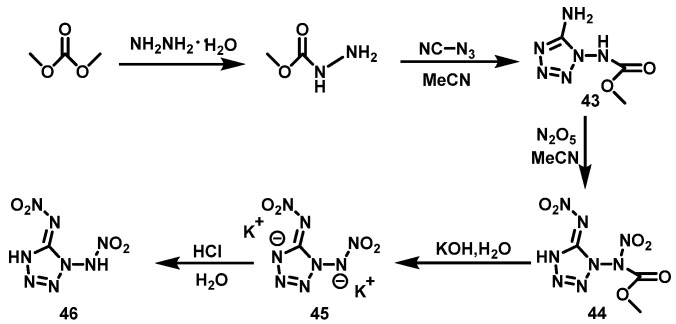
Synthesis of 1,5-di(nitramino)tetrazole [39].

Fischer et al. [39] synthesized 1-methoxycarbonyl-1,5-diaminotetrazole (43) using dimethyl carbonate via nucleophilic substitution and a ring formation reaction. After that, compound **43** was suspended in anhydrous acetonitrile at 0 °C, a solution of N_2_O_5_ in acetonitrile (MeCN) was added, and the mixture was stirred for 1 h. KOH aqueous solution was then added dropwise, the aqueous phase was separated, and the water was evaporated under high vacuum. The residue was stirred in methanol for several hours. The reaction solution was filtered, washed with methanol, and dried. The obtained solid was dissolved in 2M HCl, and 1,5-bis(nitroamino)tetrazole (46) was obtained by extraction with ethyl acetate in a yield of 50%. The synthesis route is shown in Scheme 24. The reaction has less heat generation and the temperature is easy to control. Moreover, the product separation is simple with no waste acid treatment.

(3) 100% HNO_3_ system

**Scheme 25:**
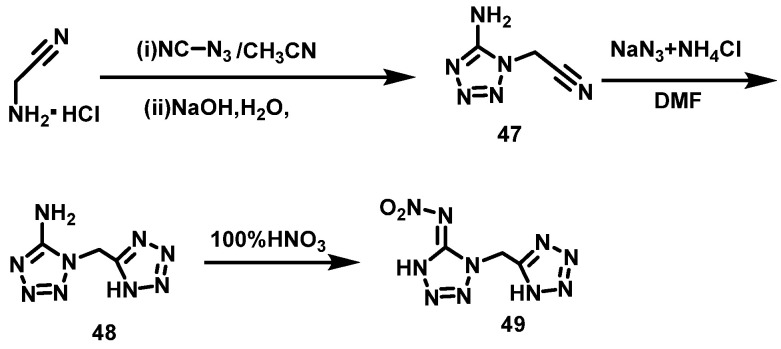
Synthesis of *N*-(1-((1*H*-tetrazol-5-yl)methyl)-1*H*-tetrazol-5(4*H*)-ylidene)nitramide [40].

Kumar et al. [40] synthesized amino 1-((1*H*-tetrazol-5-yl)methyl)-1*H*-tetrazole-5-amino (48) using aminoacetonitrile hydrochloride and cyanide azide (47) as raw materials through two-step ring formation reaction. Then, compound **2** was slowly added to 100% HNO_3_ at 0–2 °C. After stirring for 12 h at room temperature, the mixture was poured into cold water. Thus, *N*-(1-((1*H*-tetrazol-5-yl)methyl)-1*H*-tetrazole-5 (4*H*)-alkylene)nitramine (49) was obtained by extraction with ethyl acetate in a yield of 65%. The synthesis route is shown in Scheme 25.

#### 2.4.2. Nitrification of -NH_2_ Connected with Tetrazole Ring N

(1) NO_2_BF_4_ system

**Scheme 26:**
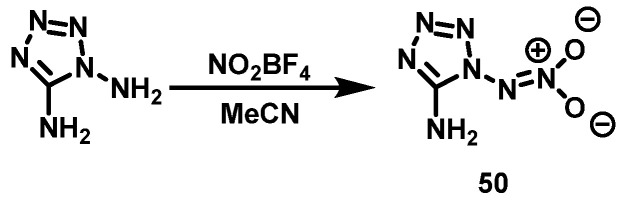
Synthesis of 5-amino-1-nitriminotetrazole [41].

Klapçtke et al. [41] dissolved 1,5-diaminotetrazole in anhydrous acetonitrile at 0 °C and added NO_2_BF_4_ to the solution with stirring. The reaction mixture was then stirred overnight at room temperature. A pale yellow solid was obtained by evaporating acetonitrile under vacuum. The solid was re-dissolved in a small amount of ethanol, and then a mixed solution of KOH and ethanol was added to precipitate potassium tetrafluoroborate. Followed by filtration, the filtrate was evaporated and 5-amino-1-nitroaminotetrazole (HDATNO_2_, 50) was obtained with a yield of 59%. The synthesis route is shown in Scheme 26. The nitrating agent, NO_2_BF_4_, is environmentally friendly and does not require waste acid treatment. The reaction conditions are mild and the temperature is easy to control. There are fewer by-products in the reaction and the selectivity of the reaction is high, but NO_2_BF_4_ is expensive and has high costs.

(2) N_2_O_5_ system

**Scheme 27:**
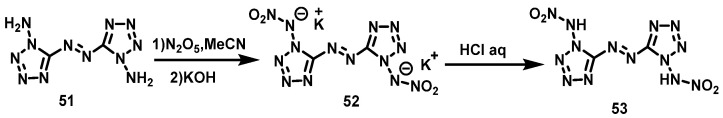
Synthesis of 1,1′-dinitramino-5,5′-azobitetrazole [42].

Fischer et al. [42] suspended 1,1′-diamino-5,5′-azobitetrazole (51) in dry acetonitrile at 0 °C and added a solution of N_2_O_5_ in cold acetonitrile dropwise. The KOH solution was added dropwise until 1,1′-diamino-5,5′-azobitetrazole was dissolved, and then the red crystal of 1,1′-dinitroammonium-5,5′-azobitetrazole dipotassium salt (52) was obtained via filtration. The yield was 62%. Subsequently, the salt 4 was dissolved in 2M HCl, and a colorless single crystal of 1,1′-dinitroammonium-5,5′-azotetrazole (53) was obtained via extraction with ethyl acetate. The synthesis route is shown in Scheme 27. This reaction uses N_2_O_5_ as a nitration system, which has higher nitration selectivity, less side reactions, superior yield, and lower equipment requirements, thereby reducing the cost of the entire process.

### 2.5. Oxadiazoles

#### 2.5.1. Nitration of -NH_2_ on 1,2,4-Oxadiazole Ring C

(1) 100% HNO_3_ system

**Scheme 28:**
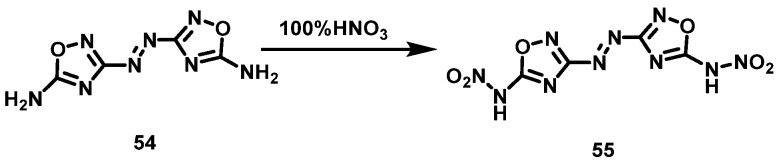
Synthesis of 5,5′-dinitramino-3,3′-azo-1,2,4-oxadiazole [43].

Tang et al. [43] slowly added 5,5′-diamino-3,3′-azo-1,2,4-oxadiazole (54) to 100% HNO_3_ at −5 °C and then slowly raised the temperature to room temperature. The mixture was stirred overnight. 5,5′-dinitroammonium-3,3′-azo-1,2,4-oxadiazole (55) was obtained via filtration and washed with TFAA, with a yield of 75%. The synthesis route is shown in Scheme 28. The reaction yield is high and the obtained product is pure without further purification.

(2) HNO_3_/Ac_2_O system

**Scheme 29:**
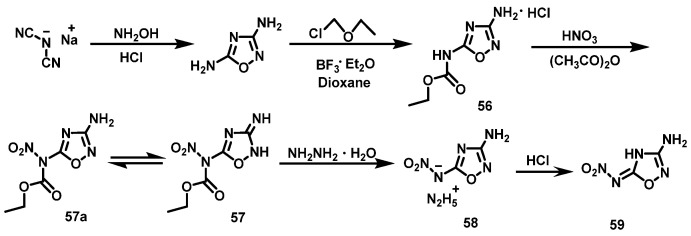
Synthesis of 3-amino-5-nitramino-1,2,4-oxadiazole monohydrate [44].

Tang et al. [44] synthesized 3-amino-5-*N*-ethoxychloroamido-1,2,4-oxadiazole hydrochloride (56) using the sodium salt of malononitrile and hydroxylamine as raw materials (Scheme 29) through a ring formation reaction and substitution reaction. HNO_3_ (100%) was slowly added to the acetic anhydride at 0 °C. Compound **56** was then added to the mixture and stirred for 1 h. Afterwards, the reaction solution was poured into ice water. The pure product of 3-amino-5-*N*-nitro-ethoxyformamido-1,2,4-oxadiazole (57) was obtained via filtration and washing with cold water. The yield was 73%. Subsequently, compound **57** was treated with a solution of hydrazine in acetonitrile to obtain 3-amino-5-nitroamino-1,2,4-oxadiazole hydrazine salt (58). 3-amino-5- nitroamino-1,2,4-oxadiazole monohydrate (59) was obtained via acidification with concentrated hydrochloric acid.

#### 2.5.2. Nitrification of -NH_2_ on 1,3,4-Oxadiazole Ring C

(1) 100% HNO_3_ system

**Scheme 30:**
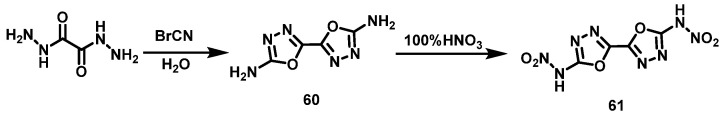
Synthesis of 2,2′-dinitramino-5,5-bi(1-oxa-3,4-diazole) [45].

Tobias et al. [45] synthesized intermediate 2,2′-diamino-5,5′-bis(1-3,4-oxadiazole) (60) using oxalodiazide and BrCN as raw materials. Compound **60** was added to 100% HNO_3_ in portions at 0 °C, and the mixture was stirred for 12 h at room temperature. 2,2′-dinitroamino-5,5-bi(1-3,4-oxadiazole) (61) was obtained by filtering and washing with water, methanol, and ether, respectively, with a yield of 84%. The synthesis route is shown in Scheme 30. In this reaction, 100% HNO_3_ is used as a nitrating agent, and the yield is greater than 80% with a high purity of the nitrated product.

#### 2.5.3. Nitration of -NH_2_ Connected with 1,2,5-Oxadiazole(Furazan) Ring C

(1) N_2_O_4_/100% HNO_3_ system

**Scheme 31:**
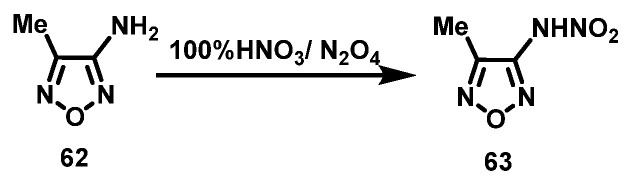
Synthesis of 3-nitroamino-4 -methylfurazan with N_2_O_4_/100% HNO_3_ system [46].

Sheremetev et al. [46] added 3-amino-4-methylfurazan (62) to a mixture of 100% HNO_3_ and 2% N_2_O_4_ at −5 °C, and stirred for 1 h. Then, the reaction mixture was heated to 20 °C and poured onto ice. 3-nitroamino-4-methylfurazan (63) was obtained via extraction with diethyl ether, washing with cold water, and drying with MgSO_4_, with a yield of 40–45%. The synthesis route is shown in Scheme 31.

(2) NO_2_BF_4_ system

**Scheme 32:**
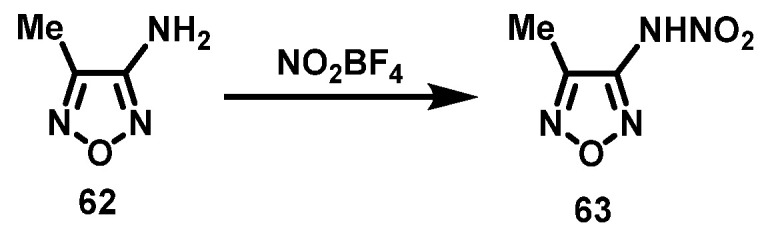
Synthesis of 3-nitroamino-4 –methylfurazan with NO_2_BF_4_ system [46].

Sheremetev et al. [46] also added 3-amino-4-methylfurazan (62) to a CH_2_Cl_2_ suspension of NO_2_BF_4_. The mixture was cooled to 0 °C, stirred for 1 h, and then the temperature was slowly raised to 5 °C. Subsequently, the reaction mixture was poured onto ice, and the resulting emulsion was extracted with diethyl ether to obtain 3-nitroamino-4-methylfurazan (63) in a 63% yield. The synthesis route is shown in Scheme 32.

(3) 100% HNO_3_ system

**Scheme 33:**
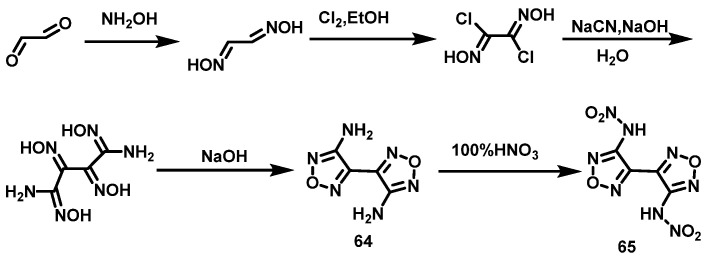
Synthesis of 3,3′-dinitramino-4,4′-bifurazane [47].

Fischer et al. [47] synthesized 3,3′-diamino-4,4′-bifurazan (64) using glyoxal and hydroxylamine by nucleophilic addition, chlorination, substitution, nucleophilic addition, and cyclization reactions. Then, compound **64** was slowly added to 100% HNO_3_ at −5 °C to 0 °C, and stirred for 45 min. The suspension was poured onto ice and 3,3′-dinitroamino-4,4′-furazan (65) was obtained by filtering and washing with ice water, with a yield of 80%. The synthesis route is shown in Scheme 33. The yield can be increased to more than 90% by using an organic solvent (such as ethyl acetate) to extract filtrate.

(4) HNO_3_/Ac_2_O system

**Scheme 34:**
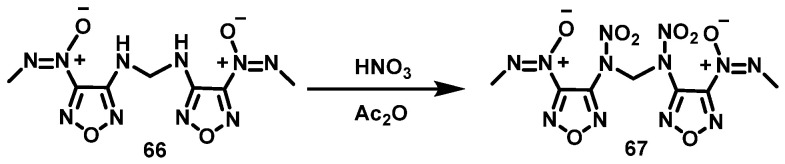
Synthesis of *N*,*N*′-dinitro-*N*,*N*′-bis[3-(methyl-NNO-azoxy)furazan-4-yl]methylenediamine [48].

Zhang et al. [48] added compound (66) in batches to a mixture of acetic anhydride and 100% HNO_3_ at 0 °C. The reaction mixture was stirred for 6 h at room temperature and then poured into ice water. *N*,*N*′-dinitro-*N*,*N*′-bis [3-(methyl-azo-nitrogen oxide) furazan-4-yl] methylene diamine (67) was obtained by filtering and washing the precipitate with ethanol in a yield of 66%. The synthesis route is shown in Scheme 34. In this nitration system, the acid anhydride can effectively reduce the oxidizing property of HNO_3_, preventing the generation of by-products. The reaction has simple operation procedures and a high yield.

(5) N_2_O_5_ system

**Scheme 35:**
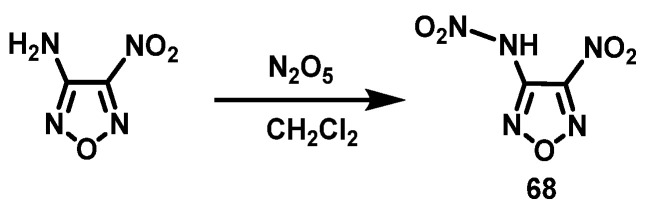
Synthesis of 3-nitramino-4-nitrofurazan [49].

Klapötke et al. [49] cooled dichloromethane (CH_2_Cl_2_) to −20 °C and added N_2_O_5_, while keeping the temperature at −20 °C. After it was completely dissolved, 3-amino-4-nitrofurazan was slowly added at −20 °C. The solution was slowly warmed up to 0–5 °C and stirred for 3 h. The solvent was removed under a constant nitrogen stream until most of the solvent was removed and 3-nitramino-4-nitrofurazan (68) was obtained in a yield of 66%. The synthesis route is shown in Scheme 35.

## 3. Nitrification of Azines as Nitrogen-Rich Heterocyclic Energetic Compounds

### 3.1. Pyridazines

#### Nitrification of H on Pyridazine Ring C

**Scheme 36:**
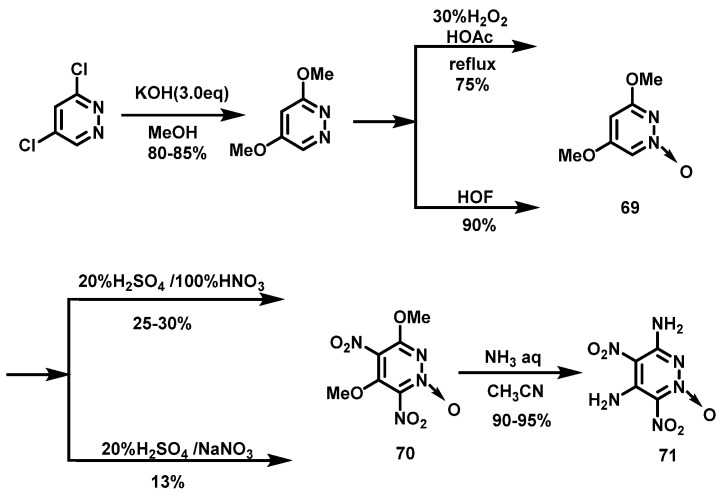
Synthesis of 3,6-diamino-4,6-dinitropyridazine-1-oxide [50].

Gospodinov et al. [50] prepared the compound 3,5-dimethoxypyridazine-1-oxide (69) using dichloropyridazine as a raw material by substitution reaction and oxidation reaction, and then used two nitration systems to obtain compound **70**. The synthesis route is shown in Scheme 36.

(1) 20% H_2_SO_4_/100% HNO_3_ system

Compound **69** was dissolved in 20% fuming H_2_SO_4_ at 5 °C, after which, NaNO_3_ was added in batches, the reaction mixture was stirred for 1 h, and was then slowly warmed up to room temperature. After that, the reaction mixture was stirred overnight at 60 °C and then poured onto crushed ice. The resulting suspension was stirred until the ice dissolved and the resulting precipitate was filtered. The crude product was dissolved in conc. H_2_SO_4_, stirred for 3 h at 60 °C, and then poured on ice and the precipitate was filtered. 3,5-dimethoxy-4,6-dinitropyridazine-1-oxide (70) was obtained after washing with ice water several times in a yield of 13%.

(2) 20% H_2_SO_4_/100% NaNO_3_ system

Compound **69** was dissolved in 20–25% fuming H_2_SO_4_ at 10 °C, and 100% HNO_3_ was added dropwise below 8 °C. The reaction mixture was first stirred at 0 °C for 1.5 h, then at room temperature for 2 h, and finally stirred at 45–50 °C for 20 h. After cooling, the reaction was poured onto crushed ice. The resulting suspension was stirred for 2 h and the obtained yellowish precipitate was filtered off and washed with water. The crude product was dissolved in conc. H_2_SO_4_ and stirred at 60 °C for 2 h. The mixture was poured onto crushed ice, filtered, and washed with ice water to obtain compound **70** in a yield of 28%. Finally, 3,6-diamino-4,6-dinitropyridazine-1-oxide (71) was synthesized by reacting compound **70** with concentrated ammonia in acetonitrile solution.

### 3.2. Pyrazines

#### Nitrification of H on Pyrazine Ring C

**Scheme 37:**
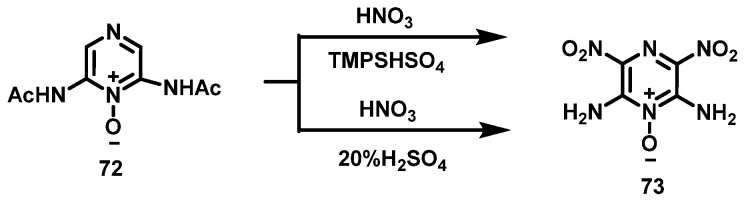
Synthesis of 2,6-diamino-3,5-dinitropyrazine-1-oxide [51].

The density of 2,6-diamino-3,5-dinitropyrazine-1-oxide (LLM-105, 73) is 1.92 g·cm^−3^ with the detonation velocity of 8516 m·s^−1^ and the detonation pressure of 35.9 GPa. It is a new explosive with excellent energy and safety performance. In the process of its preparation, there is a typical nitration reaction of H on pyrazine C, and the preparation method of LLM-105 has been improving. In 2014, Zhou et al. [51] used two nitration systems to nitrate the intermediate 2,6-diacetamidopyrazine-1-oxide (72) to obtain LLM-105. The synthesis route is shown in Scheme 37.

(1) 20% H_2_SO_4_/100% HNO_3_ system

Compound **72** was added into 20% fuming H_2_SO_4_ when the temperature was lower than 25 °C, the temperature of the mixture was controlled to be lower than 10 °C at the end of feeding, and then fuming HNO_3_ was slowly added into the mixture. Afterwards, the system was controlled to react for 1 h within 10–15 °C and then heated to room temperature for 2 h. Finally, the mixture was poured onto crushed ice, filtered, washed with water, and dried to obtain the bright yellow LLM-105 solid with the yield of 72%.

(2) 100% HNO_3_/TMPSHSO_4_ system

*N*,*N*,*N*-trimethyl-*N*-propanesulfonate-ammonium bisulfate (TMPSHSO_4_), as an ionic liquid, was dissolved in fuming HNO_3_, cooled to about 0 °C, and then compound **72** was slowly added. The reaction solution was stirred for 0.5 h; then heated to 25 °C, reacting for 1 h; and heated to 75 °C, reacting for 4 h. After the reaction was completed, the mixture was cooled to room temperature, diluted with distilled water, filtered and washed with water three times, and dried to obtain yellow LLM-105, with a yield of 68.4%. Compared with nitration with mixed acid, HNO_3_/acid ionic liquid has the advantages of simpler post-treatment and less waste acid discharge.

(3) H_2_SO_4_/95% HNO_3_ system

**Scheme 38:**
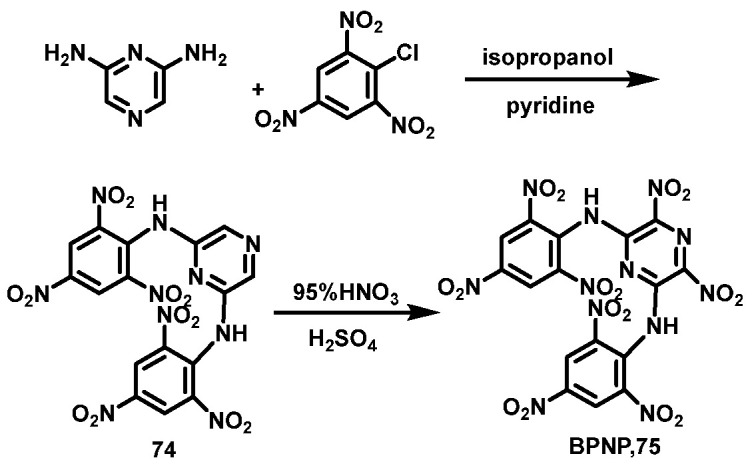
Synthesis of BPNP [52].

Liu et al. [52] synthesized 2,6-dipyramidopyrazine (74) using 2,4,6-trinitrochlorobenzene and 2,6-diaminopyrazine by condensation reaction. Next, 95% fuming HNO_3_ and concentrated H_2_SO_4_ were stirred for 0.5 h at 0 °C. After that, compound **74** was slowly added, and then the temperature was gradually raised to 50 °C for 3 h. Subsequently, the reaction solution was cooled, poured into ice water, filtered and washed with acetone, and dried in a vacuum to obtain 2,6-dipyramido-3,5-dinitro- pyrazine (BPNP, 75). The yield was 66%. The synthesis route is shown in Scheme 38.

### 3.3. Nitrification of -NH_2_ Connected with Triazine Ring C

**Scheme 39:**
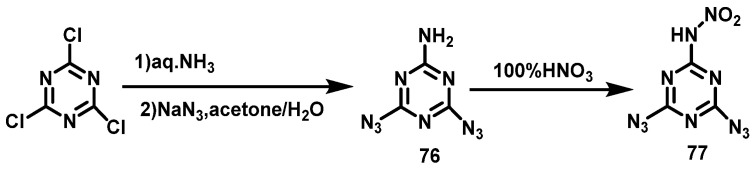
Synthesis of 2-Nitroamino-4,6-diazido[1,3,5]triazine [53].

Huang et al. [53] synthesized 6-amino-2,4-diazio[1,3,5]triazine (76) using cyanuric acid chloride with aqueous NH_3_ at 0 °C and then sodium azide in acetone/H_2_O(1:1). After that, compound (**76**) was added to HNO_3_ (100%) in batches below 5 °C, after which the reaction mixture was stirred for 30 min at 0 °C, slowly warmed up to room temperature, and stirred for another 3 h. Afterwards, the reaction mixture was poured onto ice, filtered, washed with cold water, and dried in air to obtain 2-nitroamino-4,6-diazo[1,3,5]triazine (77) in a yield of 54%. The synthesis route is shown in Scheme 39.

### 3.4. Nitrification of -NH_2_ on Tetrazine Ring C

**Scheme 40:**
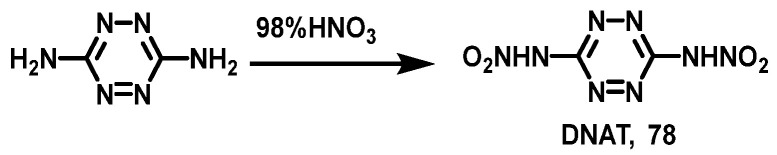
Synthesis of 3,6-dinitroamine-1,2,4,5-tetrazine [54].

Zhang et al. [54] added 3,6-diamino-1,2,4,5-tetrazine to fuming HNO_3_ at 0 °C, then gradually stirred for 1 h, filtered, and dried. 3,6-dinitroamino-1,2,4,5-tetrazine (DNAT, 78) was obtained by recrystallization from ethyl acetate with 85.0% yield. The synthesis route is shown in Scheme 40.

## 4. Conclusions

The characteristics of the used nitration agents are listed in Table 1.

The nitration methods of nitrogen-rich heterocyclic EMs of azoles (imidazole, pyrazole, triazole, tetrazole, oxadiazole) and azines (pyrazine, pyridazine, triazine, tetrazine) were reviewed. Most of the above nitro-containing nitrogen-rich heterocyclic EMs have higher density, outstanding enthalpy of formation, and excellent oxygen balance. Generally speaking, nitrogen-rich compounds with high density and enthalpy of formation always have high detonation performance. Although there are many nitration methods of nitrogen-rich heterocyclic EMs reported in the literature, there are still some problems that need to be explored and studied. The authors believe that the following aspects of research should be noted in the future:

1. Nitramino-containing nitrogen-rich EMs have higher sensitivity, and these materials can be used as primary explosives. From the viewpoint of chemical reactivity, the nitro group attached to the heterocyclic carbon adjacent to the nitrogen on the heterocyclic ring is unstable to hydrolysis. The existence of adjacent C-amino groups will improve the stability of the ring, and the lone pair of electrons on the amino nitrogen provides electrons to the ring system, thus, the sensitivity of the nitro group to hydrolysis can be significantly reduced. In addition, the presence of amino and nitro groups will boost the formation enthalpy, oxygen balance, density, and stability of the compound, thereby enhancing the detonation and safety of the compound [6,20,55]. Therefore, when designing the molecular structure of EMs, it should be designed as far as possible with compounds where nitro and amino groups cross, such as 3,6-diamino-4,6-dinitropyridazine-1-oxide (Scheme 36) and 2,6-diamino-3,5-dinitropyrazine-1-oxide (LLM-105) (Scheme 37);
2. Applying organic synthesis technologies, such as ultrasound and microwave, to the nitration process of nitrogen-rich heterocyclic energetic compounds to shorten the time and improve the overall yield is imperative;
3. In view of the traditional methods for synthesizing nitro-containing heterocyclic energetic compounds, including a series of problems brought about by the application of oleum sulfuric acid and fuming nitric acid, it is necessary to continue research so as to discover new nitrification methods to adapt to new needs, especially paying more attention to the development of some low-toxic, cheap, efficient, and environmentally friendly nitrification strategies to adapt to the implementation of sustainable development strategies and the practical application of green chemistry.

## Figures and Tables

**Table 1:** Characteristics of different nitrification systems.

Nitrification System	Characteristic
HNO_3_	Cheap. To nitrate different azole rings, different concentrations of HNO_3_ are required.
HNO_3_/H_2_SO_4_	The nitration ability is stronger than that of HNO_3_, and it is often used for the nitration of a variety of azole compounds. According to the structural characteristics of different azole rings and the difficulty of the aromatic electrophilic substitution reaction, a suitable ratio of HNO_3_/H_2_SO_4_ should be selected for nitration. The reaction conditions are mostly mild, the required temperature range is generally from 20 °C to 100 °C, the reaction time is about 1 h to 2.5 h, and the yield can reach about 80% or higher. For some azole substrates with relatively inert reaction activity (such as the existence of strong electron-withdrawing group), the reaction rate is generally slow and the reaction time needs to be extended to 10–48 h.
HNO_3_/Ac_2_O	Compared with the nitrating reagent HNO_3_/H_2_SO_4_, the HNO_3_/Ac_2_O mixture has a weaker nitrification ability, but the acid anhydride can effectively reduce the oxidation of HNO_3_.
NO_2_BF_4_	An environmentally friendly nitrating reagent without waste acid treatment. The reaction condition is relatively mild, and the temperature is easy to control. It is non-oxidizing and has high reaction selectivity. It can be used for the aromatic electrophilic nitration reaction of various azole rings, acetonitrile is often used as the solvent, the reaction time is generally 10 h, and the yield is 30–50%.
N_2_O_5_	N_2_O_5_ is a green nitrification reagent that can be used for the nitration of sensitive compounds. Compared with nitration reagents such as HNO_3_, HNO_3_/H_2_SO_4_, HNO_3_/acid anhydride, it has the following advantages: no need for waste acid treatment, the reaction has less heat release and the temperature is easy to control, the post-treatment is simple as only the solvent needs to be evaporated, the reaction is usually carried out with CH_2_Cl_2_ as solvent, the temperature is generally from 20 °C to 100 °C, and the yield is about 90%.
HNO_3_/P_2_O_5_	In this system, P_2_O_5_ is not only a dehydrating agent, but also a nitrification accelerator. The nitration system is suitable for the nitration of aromatics as well as amines.
Nitrate	Under catalysis, some nitrates can also act as nitrating reagents, which are mainly used in the synthesis of some nitropyrazole compounds. Their nitrating capacities are as follows: Bi(NO_3_)_3_ > AgNO_3_ > KNO_3_ > NaNO_3_ > NH_4_NO_3_ > Pb(NO_3_)_2_ > Ba(NO_3_)_2_.

## Data Availability

Not applicable.
